# Dr. Robert Wadsworth

**Published:** 1913-11

**Authors:** 


Dr. Robert Wadsworth, Hahnemann of Philadelphia, 1876, died October 17, in Rochester, suddenly, while making a professional call.

